# Integration of HIV in the Human Genome: Which Sites Are Preferential? A Genetic and Statistical Assessment

**DOI:** 10.1155/2016/2168590

**Published:** 2016-05-12

**Authors:** Juliana Gonçalves, Elsa Moreira, Inês J. Sequeira, António S. Rodrigues, José Rueff, Aldina Brás

**Affiliations:** ^1^Centre for Toxicogenomics and Human Health (ToxOmics), Genetics, Oncology and Human Toxicology, Nova Medical School/Faculdade de Ciências Médicas, Universidade Nova de Lisboa, Rua Câmara Pestana 6, 1150-008 Lisbon, Portugal; ^2^CMA, Department of Mathematics, Faculty of Sciences and Technology, Universidade Nova de Lisboa, Campus Caparica, 2829-516 Caparica, Portugal

## Abstract

Chromosomal fragile sites (FSs) are loci where gaps and breaks may occur and are preferential integration targets for some viruses, for example, Hepatitis B, Epstein-Barr virus, HPV16, HPV18, and MLV vectors. However, the integration of the human immunodeficiency virus (HIV) in Giemsa bands and in FSs is not yet completely clear. This study aimed to assess the integration preferences of HIV in FSs and in Giemsa bands using an* in silico* study. HIV integration positions from Jurkat cells were used and two nonparametric tests were applied to compare HIV integration in dark versus light bands and in FS versus non-FS (NFSs). The results show that light bands are preferential targets for integration of HIV-1 in Jurkat cells and also that it integrates with equal intensity in FSs and in NFSs. The data indicates that HIV displays different preferences for FSs compared to other viruses. The aim was to develop and apply an approach to predict the conditions and constraints of HIV insertion in the human genome which seems to adequately complement empirical data.

## 1. Introduction

Giemsa staining has long been used for identifying individual human chromosomes. Giemsa dark and light bands are generally thought to correspond to GC-poor and GC-rich regions, respectively. Giemsa light bands are gene-rich and contain most housekeeping genes as well as a large number of CpG islands, whereas Giemsa dark bands are gene-poor and preferentially contain tissue-specific genes. Hence, light bands are transcriptionally more active when compared to dark bands and also have an open chromatin configuration which together with the high content in GC can have an important role in provirus integration [1]. Giemsa bands are also related to functional nuclear processes such as replication. For example, DNA replication timing during cell cycle differs between both; light bands are early-replicating, whereas dark bands are late replicating. Giemsa bands are also related to chromatin structures as the chromatin in dark bands is more condensed than in light bands during both metaphase and interphase [2]. Another difference between these two Giemsa bands is that the DNA of Giemsa dark bands are located at the nucleus periphery [3] while the DNA of Giemsa light bands is in the interior of the nucleus [4].

FSs are hereditary loci of human chromosomes susceptible to occurrence of breaks, gaps, or rearrangements when under stress conditions or treated with specific chemical agents [5–7]. According to the frequency of their distribution in the human population, FSs can be divided in two distinct groups: common fragile sites (CFSs) present in all individuals and rare fragile sites (RFSs) that are present in less than 5% of the population, and these two groups can also be subdivided according to the inducing agent [8–10]. Both types of FSs have the capacity to form secondary structures that can interfere with elongation in replication [11] or even cause failure in chromatin condensation [12]. FSs are also involved in sister chromatid exchanges [7] translocations and deletions [13] and in intrachromosomal gene amplifications [14]. CFSs are very unstable regions because they contain sequences of high flexibility [15] and are regions of late replication [5] and also correspond to transition regions in replication timing [16]. Several authors have shown that fragile sites are preferential integration targets for some viruses, for example, Epstein-Barr virus [17] and human papillomaviruses HPV16 and HPV18 [18]. Recently, Christiansen et al. showed that transcriptionally active regions and FSs are the preferred targets for chromosomal HPV integration in cervical carcinogenesis [19].

The human immunodeficiency virus (HIV) is a retrovirus whose stable integration in the human genome is essential for completing its life cycle [20, 21]. The virus binds to the membrane receptors of host cells to enter the cytoplasm [22]. The RNA genome of HIV is converted into DNA by the reverse transcriptase (RT) enzyme [23] that is transported to the nucleus. Viral integration into the DNA occurs in three steps, (i) processing [24]; (ii) joining [25]; and (iii) postintegration repair [26]. After integration, transcription occurs followed by translation in the host cytoplasm. There are two different types of viruses: HIV-1, identified first in 1983 [27], and HIV-2, later discovered in 1986 [28]. Viral integration in human cells can affect gene expression, leading to molecular and epigenetic alterations, and can even activate oncogenes [22]. Thus knowledge of viral integration sites is important to understand their biological effects. Schröder et al. concluded that integration sites of HIV are not randomly distributed in the human genome, but in regional hotspots [29]. The same group also found that integration sites are related to gene-rich sites which can allow a more efficient expression of the viral genome. Moreover, according to Debyser et al. [30] each retroviral family integrates near a unique and specific subset of genomic features. HIV integration site selection is related to the pathway and efficiency of nuclear translocations [31].

Retroviruses can be used as vectors in gene therapy since they can integrate stably in the host genome [30, 32] and they have the capacity to introduce genetic material in target cells [33]. Additionally, analysis of the integration process is important in HIV-induced disease. Therefore, it is important to understand the integration preferences of HIV since knowledge of the integration sites in the human genome can help, for example, to choose gene-delivery vectors [29].

The integration preferences of HIV in light or dark bands and in FSs are not clear. Thus, our main objective was to study the HIV integration preferences in Giemsa bands and in FSs using bioinformatics and statistical analyses. More specifically we aimed to understand the integration preferences of HIV-1 isolated from Jurkat T cells in Giemsa bands and FSs by the use of an* in silico* approach based on statistical analysis which may complement laboratorial studies and predict HIV constraints and preferences of integration in the human genome.

## 2. Methods

### 2.1. Data

#### 2.1.1. HIV Integration Sites

For the HIV-1 isolated from Jurkat T cells Wang et al. have supplied the exact position of the integration sites [34].

#### 2.1.2. Giemsa Bands and FSs

The positions of Giemsa bands used in this study were obtained* in silico* by Niimura and Gojobori [2], available from the Center for Information Biology and DNA Data Bank of Japan, National Institute of Genetics, in http://yosniimura.net/research/coordinates.html.

Regarding FSs, the human genome was divided in FRs and NFRs, according to their positions. A list of FSs was obtained from Mrasek et al. [6] and completed with FSs from Lukusa and Fryns [8]. Two consecutive bands associated with FSs were grouped to form a fragile region (FR) and a region between two FRs was considered a NFR [35, 36]. The Y chromosome was not considered because it does not have well defined FSs; there is only a possibility of existing one FS [37].

### 2.2. Statistical Analysis

Integration sites of HIV-1 were colocated with Giemsa dark bands and classified in two groups:* yes* if they colocalized and* no* if they did not. Some integrations occurring in the centromeres and short arm of the acrocentric chromosomes were excluded from the total. Thus for HIV-1 we have integrations in 24 chromosomes in a total of 42912 integration sites. Then a measure was calculated to determine in which type of band the virus integrates. The measure is designated as* integration intensity number* and is given by

(1) idark band=nyesldark band;ilight band=nnollight band,

where *n*_yes_ represent the number of viral integrations in dark bands, *l*_dark band_ is the length of the dark bands, *n*_no_ is the number of viral integrations in light bands, and *l*_light band_ is the length of the light bands.

After the calculations of the measure, one pair (*x*, *y*) was obtained for each chromosome, where *x* represents the measurement value in dark bands and *y* the measurement value in light bands. To compare dark bands with light bands graphical representations for the measure were constructed where each point represents one chromosome. In order to see the preferences of integration of the virus, in each graphic the line *y* = *x* was represented that allows the visualization of the number of chromosomes in which *y* > *x* and *y* < *x*, in other words, if the virus preferentially integrated in dark or in light bands. In order to statistically verify the results obtained graphically two nonparametric tests were applied that enable us to compare two dependent samples, the Sign and the Wilcoxon tests [38]. In both tests a significance level of 1% was used to test our hypothesis.

The same methodologies were applied in FSs, also classifying the integrations in two groups:* yes* if they colocalized with a FR and* no* if they colocalized with a NFR. Integrations of HIV-1 for the 23 chromosomes were obtained, in a total of 44150 integrations sites.

## 3. Results and Discussion

### 3.1. Preferential Integration of HIV-1 in Giemsa Light Bands

**Figure 1:**
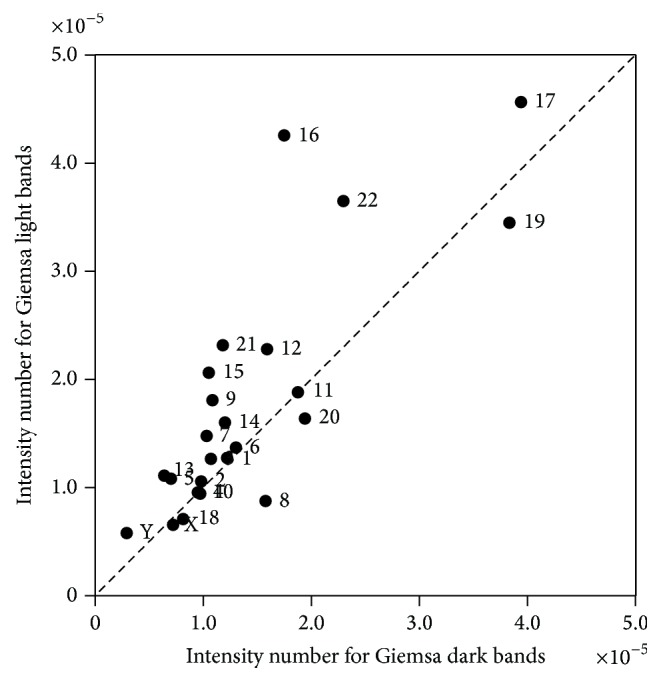
Integration of HIV-1 isolated from Jurkat T cells in Giemsa dark bands versus Giemsa light bands. Graphical representation of the results for the* intensity number.* Each point represents a chromosome whose coordinates are the (*x*, *y*) pairs obtained in the measure calculations. The statistical analysis indicates that the virus integrates preferentially in Giemsa light bands (Wilcoxon test, *T*_obs_: 59; *T*_critic_: 69).

The* intensity number* for the HIV-1 isolated from Jurkat T cells was calculated and the graphical representation is presented in Figure 1 for Giemsa bands. For the Wilcoxon test, *T*_obs_ (59) was lower than *T*_critic_ (69), so the hypothesis of equal intensity at 1% level can be clearly rejected. It means that the intensity of integration is not equal in the two types of bands and it can be concluded that HIV-1 isolated from Jurkat T cells integrates with more intensity in Giemsa light bands.

Our* in silico* results obtained for Giemsa bands indicate that HIV-1 isolated from Jurkat T cells integrates preferentially in light bands, which have a high content in GC [2]. These results are in line with previous studies which reveal that HIV favours integration in transcriptionally active units [34, 39, 40] which are associated with regions of high GC content and high gene density [24]. When integrated in transcriptionally active regions this ensures viral gene transcription [41, 42]. The virus has a limited time to replicate [24] since T cells infected with HIV have a very short half-time [43]. Thus the virus has to integrate in regions that accelerate its transcription. Regions that are more transcriptionally active allow an efficient maintenance of the replication cycle of the virus since they permit a higher provirus transcription [44], increasing viral gene expression [34].

Elleder et al. also found that HIV integrates preferentially in Giemsa light bands and in regions with open chromatin which favours integration [44], since the IN enzyme of HIV-1 interacts with components of the chromatin remodeling complex [45]. The interaction with LEDGF/p75 accounts for the karyophilic properties and chromosomal targeting of HIV-1 IN [46]. Nevertheless, one must note that virus integration in transcriptionally active regions with an open chromatin conformation, that is, light bands, could also be a by-product of the integration of HIV-1 integrase with components of the chromatin remodeling complex, which could impact on our interpretation of HIV integration site preference.

Marini et al. also revealed that the cellular genes that are targeted by HIV-1 are enriched in open chromatin marks associated with the nuclear complex pore that are constituted by nucleoporins which participate in HIV-1 transcriptional regulation [47]. This report supports our results for the integration in Giemsa light bands that have a less condensed chromatin than Giemsa dark bands. Moreover, the same group of authors also found that the areas of open chromatin that are targeted by the HIV-1 preintegration complex are those proximal to the nuclear pore. HIV-1 could also have a preference for Giemsa light bands because they have active genes [48]; thus the preference for active gene regions may have been developed to favour HIV gene expression after integration [29]. Another feature that supports our results is that light bands possess high levels of histone acetylation, namely, of histones H3 and H4, which enable access of transcription factors [48, 49]. In this regard Wang et al. demonstrated that the frequency of integration of HIV is associated with epigenetic modifications including H3 and H4 acetylation [34].

### 3.2. FSs Are Not Preferential Integration Targets for HIV-1

**Figure 2:**
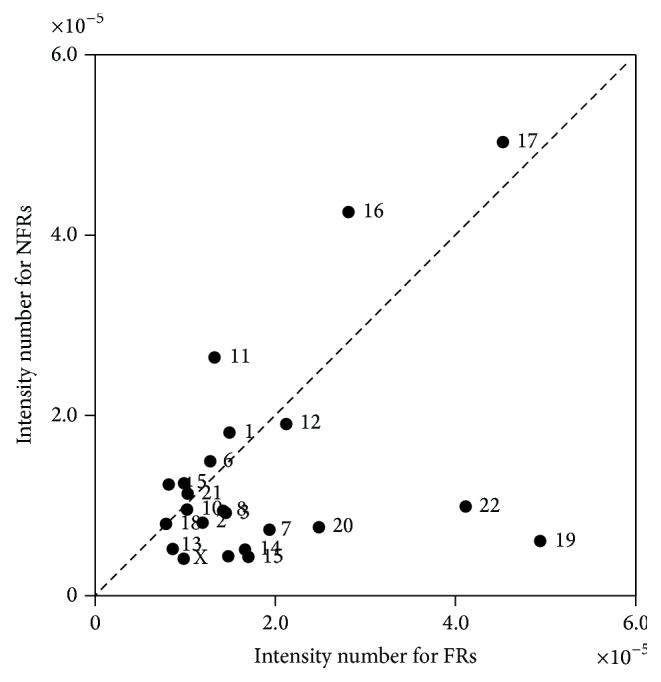
Integration of HIV-1 isolated from Jurkat T cells in FRs versus NFRs. Graphical representation of the results for the* intensity number.* Each point represents a chromosome whose coordinates are the (*x*, *y*) pairs obtained in the measure calculations. The statistical analysis indicates that the virus integrates with equal intensity in FRs and in NFRs (Sign test, *p* value: 0.202, and Wilcoxon test, *T*_obs_: 82; *T*_critic_: 62).

Figure 2 shows the result of* intensity number* for the HIV-1 virus isolated from Jurkat T cells in FRs. For the Sign test a nonsignificant *p* value of 0.202 was obtained. The Wilcoxon test resulted in a *T*_obs_ of 82 and a *T*_critic_ of 62 which leads us to accept the hypothesis of equal integration intensity. Thus, HIV-1 isolated from Jurkat T cells integrates with equal intensity in FRs and NFRs.

In the methodology followed, two consecutive bands associated with FSs were grouped to form a FR and a region between two FRs was considered a NFR. We verified that HIV-1 isolated from Jurkat cells integrates with equal intensity in FRs and in NFRs. Other known viruses, such as Hepatitis B, Epstein-Barr virus [17], HPV16, and HPV18 [18], integrate more in FSs. These differences between HIV and the other viruses may be explained by the different phases of the cell cycle in which the virus enters in human cells. Pyeon et al. verified that progression in cell cycle through mitosis is critical to HPV infection [50] while HIV can infect nondividing cells [24, 31]. The fact that HIV does not have a tendency to integrate in FRs could be related to the structure of the FSs which are vulnerable to DNA breaks [51], which may not facilitate viral integration or the conclusion of viral replication. Moreover, FSs have the tendency to form secondary structures, which interfere with replication [11] thus hampering viral integration and replication. Genomic instability is another characteristic of FSs [7] which does not favour viral integration as it needs to integrate its genome stably in hosts to complete its life cycle [21]. HIV integrates more in regions with GC content which is not the case of FSs that are predominantly constituted by AT [15, 52].

In both graphics for Giemsa bands and for FSs we found that there were some chromosomes that differ from the rest, for example, chromosome 17. This result is in agreement with the data by Soto et al. [53]. This chromosome is rich in protein coding genes and has a high CG content which indicates a high gene density, besides being rich in SINEs [54].

## 4. Conclusion

Our aim was to develop and apply an approach intended to predict the conditions and constraints of HIV-1 insertion in the human genome. At the present stage, our approach seems to adequately predict most of the conditions unravelled by empirical data but is still not exempt from weaknesses. We concluded that HIV-1 isolated from Jurkat T cells integrates with more intensity in Giemsa light bands and with equal intensity in FRs and NFR. Our work is based on statistical analysis which complements laboratorial studies. Other factors such as the cell cycle phase and the cellular type that act* in vivo* could also influence the integration site selection of the virus. Moreover, the distribution of integration sites may be altered* in vitro* due to repeated cell division and selection for certain clones. Nevertheless, our data are in line with previous reports and may contribute to the understanding of viral integration in HIV disease and gene therapy strategies. The presented* in silico* approach offers promise of useful application.
